# Supplementary material for: Identification of photoperiod-induced specific miRNAs in the adrenal glands of Sunite sheep (Ovis aries)
Source: Front Vet Sci. 2022 Jul 22;9:888207. doi: 10.3389/fvets.2022.888207 (PMC9354845; doi:10.3389/fvets.2022.888207)
Supplement: Supplementary file 1 [file Data_Sheet_1.ZIP › Supplementary file/miRDeep2 file.zip!/miRDeep2 file/Know_Structure/SP42A2/oar-mir-23a.pdf]

miRBase precursor : oar-mir-23a
Total read count : 71200
oar-miR-23a read count : 35638
oar-miR-23b read count : 35559
remaining reads : 3

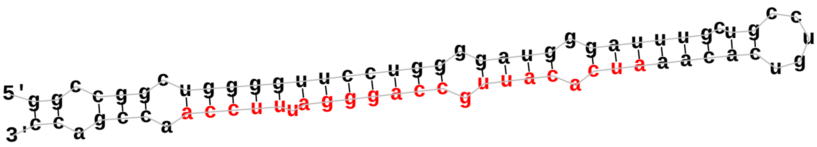

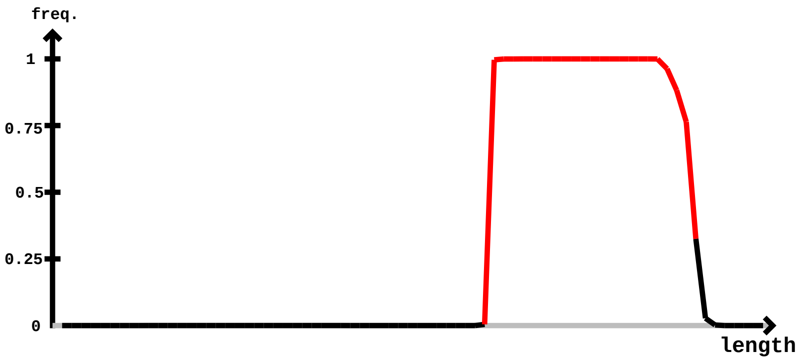


```
                                            oar-miR-23a
                                            oar-miR-23b
5'- ggccggcugggggguuccuggggaugggauuugcugccugucacaaaucacauugccagggauuuccaaccgacc   -3'    exp
    ((.(((.((((((((((((.((((.((((((.((.....)))))))).)))).))))))).))))).))).))   reads     mm      sample
    ........gggguuccuggggaugggauu.............................................   2         0       seq
    ........gggguuccuggggaugggauuu............................................   1         0       seq
    ............................................aaucacauugccagggauu...........   6         0       seq
    ............................................aaucacauugccagggauuu..........   17        0       seq
    ............................................aaucacauugccagggauuuc.........   24        0       seq
    ............................................aaucacauugccagggauuucc........   83        0       seq
    ............................................aaucacauugccagggauuucca.......   28        0       seq
    ............................................aaucacauugccagggauuuccaa......   1         0       seq
    .............................................aucacauugccagggau............   9         0       seq
    .............................................aucacauugccagggGu............   1         1       seq
    .............................................aucacauugccagggaC............   1         1       seq
    .............................................aucacauugcUagggauu...........   2         1       seq
    .............................................aucacauugccUgggauu...........   1         1       seq
    .............................................aucacauugccagggauu...........   1292      0       seq
    .............................................aucacauugccagggauuu..........   2853      0       seq
    .............................................aucacauugccagggauuuc.........   4184      0       seq
    .............................................aucacauugccagggauuucc........   15517     0       seq
    .............................................aucacauugccagggauuucca.......   10537     0       seq
    .............................................aucacauugccagggauuuccaa......   891       0       seq
    .............................................aucacauugccagggauuuccaac.....   70        0       seq
    ..............................................ucacauugccagggauu...........   6         0       seq
    ..............................................ucacauugccagggauuu..........   5         0       seq
    ..............................................ucacCuugccagggauuu..........   1         1       seq
    ..............................................ucacauugccagggauuuc.........   11        0       seq
    ..............................................ucacauugccagggauuucc........   35        0       seq
    ..............................................ucacauugccagggauuucca.......   33        0       seq
    ..............................................ucacauugccagggauuuccaa......   10        0       seq
    ..............................................ucacauugccagggauuuccaac.....   9         0       seq
    ...............................................cacauugccagggauuucca.......   1         0       seq
    ................................................acauugccagggauuuc.........   1         0       seq
    ................................................acauugccagggauuucc........   4         0       seq
    ................................................acaAugccagggauuucc........   1         1       seq
    ................................................acauugccagggauuucca.......   4         0       seq
```
